# Genetic diversity and population structure of endangered plant species *Anagallis foemina* Mill. [*Lysimachia foemina* (Mill.) U. Manns & Anderb.]

**DOI:** 10.1007/s12298-020-00839-6

**Published:** 2020-07-07

**Authors:** Ewa Kwiecińska-Poppe, Małgorzata Haliniarz, Sylwia Sowa, Edyta Paczos-Grzęda

**Affiliations:** 1 Department of Herbology and Plant Cultivation Techniques, University of Life Sciences in Lublin, Lublin, Poland; 2 Institute of Plant Genetics, Breeding and Biotechnology, University of Life Sciences in Lublin, Lublin, Poland

**Keywords:** Genetic similarity, ISSR, Molecular markers, *Primulaceae*

## Abstract

*Anagallis foemina* L. [syn. *Lysimachia foemina* (Mill.) U. Manns & Anderb.] is an annual, segetal weed from the family *Primulaceae*, recognized as a very rare and endangered species in many European countries. The rare occurrence of this species is associated with the specificity of the habitats in which it occurs. Knowledge of genetic diversity within and between rare species populations is a crucial step for investigating the causes of extinction as well as developing effective conservation strategies. The current study undertakes the assessment of the genetic variation and population structure of *Anagallis foemina* L. specimens collected in south-eastern Poland, Volhynian Polesie and West Volhynian Upland based on inter-simple sequence repeats (ISSR) polymorphism. Twenty ISSR primers amplified 374 DNA fragments, of which 79% were polymorphic. The polymorphic information content values ranged from 0.230 to 0.430 with an average of 0.344. An average genetic similarity calculated based on Dice algorithm between all analysed samples was 0.635 (0.28–1.00). The AMOVA study found a significant difference (Φ_pt_ = 0.88, *P* = 0.001) between *Anagallis* L. genotypes gathered in Volhynian Polesie (VP) and West Volhynian Upland (VU). Analysis indicated, that 89% of the variation existed among groups and 11% within groups. UPGMA analyses grouped *A. foemina* samples into 2 clearly separated clusters. The plants of the same geographic origin were grouped together. Principal coordinates analysis (PCoA) as well as STRUCTURE also grouped samples in consistence with the collection site, indicating close genetic affinity of plants from the same location. The observed results are typical for fragmented and isolated populations of rare species. Isolation of a small population leads to a decrease in internal genetic variation and to an increase of variation among them. In that case, the conservation of populations from each regional cluster is important to preserve biodiversity.

## Introduction

According to the taxonomy established by Pax and Knuth (1905) and modified by Hu (1994), Källersjö et al. (2000), Stahl and Anderberg (2004), tribe Lysimachieae (*Primulaceae*) included six genera: *Lysimachia*, *Anagallis*, *Trientalis*, *Glaux*, *Asterolinon*, and *Pelletiera*. However, based on cpDNA as well as rDNA sequence studies (Banfi et al. 2005; Manns and Anderberg 2005, 2007a, 2009; Yan et al. 2018), this traditional classification has been changed and all genera merged into *Lysimachia*. Currently, *Anagallis* is accordingly treated as a group nested within *Lysimachia* L. genus (Källersjö et al. 2000) placed in *Primulaceae* (China Phylogeny Consortium 2016).

*Anagallis* group comprises 31 species of small herbs with pink, white, and sometimes red or blue flowers, with a majority narrowly distributed in tropical areas. Only a few species (i.a. *A. monelli* or *A. foemina*) occur in Mediterranean region or in Europe, including *A. arvensis*, a weed that has a more widespread global distribution (Anderberg and Ståhl 1995).

In Poland, there are two species of *Anagallis*, *A. arvensis* [syn. *Lysimachia arvensis* (L.) U. Manns & Anderb.] and *A. foemina* [syn. *Lysimachia foemina* (Mill.) U. Manns & Anderb]. The first is common in segetal and ruderal communities throughout the country and increases in numbers (Kornaś 1962; Staniak et al. 2017). The other, *A. foemina*, is a species, which sporadically and only locally occurs in agrophytocoenoses or in uncultivated areas on calcareous soils (Fijałkowski 1978; Haliniarz and Kapeluszny 2014).

*A. foemina* is an archeophyte which was introduced to Poland before the end of the 15th century (Fijałkowski 1978). This species grows mainly in extensive cereal or root crops (Kornaś 1962), less often on roadside and rubble (Fijałkowski 1978). The rare occurrence of *A. foemina* is associated with the specificity of the habitats in which it occurs. It is found in sunny, warm and dry places (Kornaś 1962). It grows on calcium rich soils, most often on relatively dry heavy chalky rendzinas produced on chalk marls and tertiary gypsum sediments (Fijałkowski 1978). The number of *A. foemina* populations is constantly decreasing (Siciński and Sieradzki 2010) and is reported as a species at risk of extinction in many European countries (Brütting 2013; Kolárová et al. 2013). The species was placed on the national red list of Polish plants and fungi in the category of endangered species (Kaźmierczakowa et al. 2016). On the Volhynian Polesie and West Volhynian Upland according to Kucharczyk (2004), *A. foemina* belongs to critically endangered species, but based on Haliniarz and Kapeluszny (2014) as well as Cwener et al. (2016) studies, this species is exposed to extinction.

Thus far, molecular analysis of *A. foemina* concerned mainly of intrageneric relationships assessment within *Anagallis,* based on rDNA or cpDNA sequence polymorphisms, so were concentrated on the restricted parts of a genome (Banfi et al. 2005; Manns and Anderberg 2007b, 2009; Yan et al. 2018). The aim of our study was primarily to investigate the genetic diversity within endangered species *A. foemina* using samples collected in south-eastern Poland employing ISSR (inter simple sequence repeats) method developed to identify DNA polymorphism in the proximity of microsatellites. Invaluable information on the basis of this studies regarding endangered species protection, not only in Poland, but also in European countries is expected.

## Materials and methods

### Plant material

*Anagallis foemina* Mill. [synonyms: *Anagallis femina* Will., *Anagallis coerulea* Schreb., *Anagallis arvensis* subsp. *caerulea* (Schreb.), *Lysimachia foemina* (Mill.) U. Manns & Anderb] is a species with an erect, branched stem, growing up to 20 cm tall. Its dark green leaves, 5–20 mm long, are narrow-ovate, margin usually without glands, pointed. The upper leaves are often narrower than the lower ones and placed opposite on the stem. The flowers are 8 mm in diameter, five-petal, blue, red at the base. The petals of the crown do not overlap each other deeply, they have a few, non-glandular cilia 6 × 3.5 mm. Flower stalks are set individually in the leaf axils, during flowering, they are shorter or as long as the supporting leaves. It blooms and produces seeds from May to October. The fruit is a spherical, multi-seed bag (Rutkowski 2006). The number of *A. foemina* chromosomes is usually 2n = 40, but polyploidy is thought to be present in some populations (Clapham et al. 1987). It is an insect-pollinated and self-pollinating species. Its fertility is estimated at 200–300 seeds per plant, while the mass of a thousand seeds ranges from 0.6 to 0.7 g (Brütting 2013; Brütting et al. 2012a, b).

**Fig. 1:**
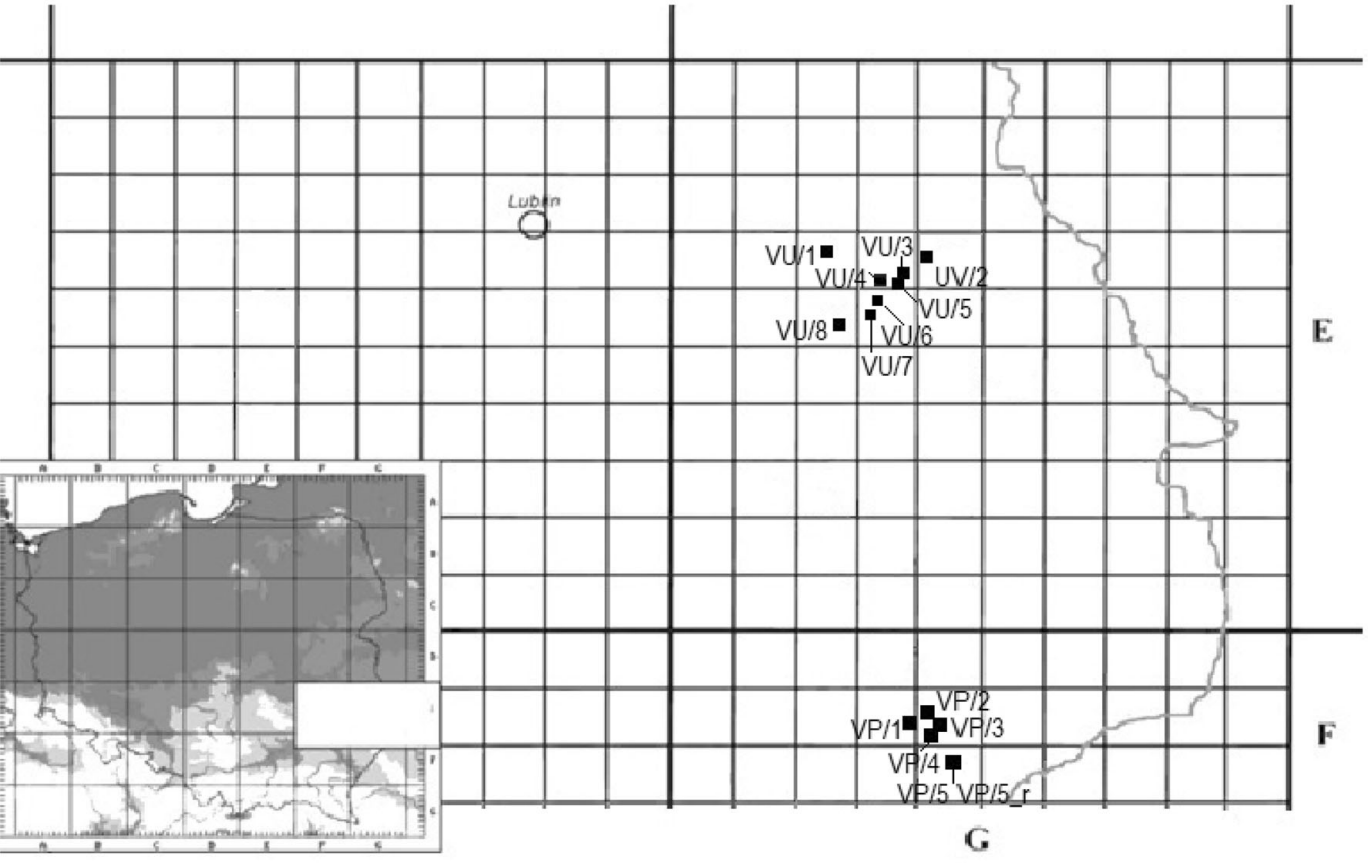
Geographical distribution of collection sites of *Anagallis foemina* Mill. in south-eastern Poland

Plant material for genetic analyses was acquired in the years 2014–2016 (from June to July) from sites located in the fields of south-eastern Poland (Table 1, Fig. 1). Plants were collected from segetal communities, such as: winter wheat, spring wheat, spring barley, winter triticale, and sugar beet. A total of 14 *Anagallis foemina* L. samples as well as one *Anagallis arvensis* L. (as an outgroup for genetic analysis) were collected. Species affiliation was made through general characters for the species and the location. From each site, leaves were picked from 5 randomly selected plants. The exact geographical position of the sites was determined using a GPS device (Table 1).

**Table 1:** Geographical origin of *Anagallis* L. genotypes used in the study

Sample	Geographical coordinates		Geographical region
	Latitude	Longitude	
VU/1	51.1822	23.3660	West Volhynian Upland (VU)
VU/2	51.1517	23.5797	
VU/3	51.1160	23.5310	
VU/4	51.1145	23.5189	
VU/5	51.1152	23.5302	
VU/6	51.0983	23.4448	
VU/7	51.0825	23.4273	
VU/8	51.0585	23.3913	
VU/9	50.9739	23.7020	
VP/1	50.4312	23.4983	Volhynian Polesie (VP)
VP/2	50.4312	23.5191	
VP/3	50.4100	23.5405	
VP/4	50.4033	23.5445	
VP/5	50.3677	23.5867	
VP/5_r	50.3677	23.5867	

Total genomic DNA was extracted from the leaf tissue of material frozen in liquid nitrogen using GeneMATRIX Plant & Fungi DNA Purification Kit (EURx) and purified with Anti-Inhibitor Kit (A&A Biotechnology). DNA integrity and quality were evaluated by electrophoresis on 1.5% agarose gel. The DNA concentration was determined with NanoDrop2000 spectrophotometry and normalized to 100 ng/μL.

### Genotyping

PCR reactions were carried out in a 12 µL volume of a mixture containing 20 ng of template DNA, 1 × PCR Master Mix buffer (0.05 U/μL *Taq* DNA polymerase, 4 mM MgCl_2_, 0.4 mM of each dNTP) (Thermo Fisher Scientific) and 0.6 nM of primer (Table 2). The amplification protocol was performed according to the ISSR (Inter Simple Sequence Repeat) method, described by Zietkiewicz et al. (1994). Following temperature profile was used: 95 °C for 7 min (pre-denaturation); 3 cycles of 95 °C for 30 s, 54 °C for 45 s, and 72 °C for 2 min; 3 cycles of 95 °C for 30 s, 53 °C for 45 s, and 72 °C for 2 min; 32 cycles of 95 °C for 30 s, 52 °C for 45 s, and 72 °C for 2 min; and a final extension of 72 °C for 7 min.

PCR products were separated on 1.5% agarose gel containing 5 μg/ml EtBr in 1/TBE Buffer (90 mM Tris–borate, 2 mM EDTA, pH 8.0). To establish molecular weight of amplification products, Gene Ruler™ 100 bp Plus DNA Ladder was used. Fragments were visualized under UV transilluminator and photographed.

**Table 2:** List of ISSR primers used in the study and values of markers polymorphism indices: TNF total number of fragments, NPF number of polymorphic fragments, PPF percentage of polymorphic fragments and PIC polymorphism information content

Name	Sequence 5′ → 3′	TNF	NPF	PPF (%)	PIC
sr16	(GA)_8_C	18	11	61	0.281
sr17	(GA)_8_YC	13	10	77	0.353
sr28	(TG)_8_G	23	22	96	0.422
sr31	(AG)_8_YC	29	22	76	0.348
sr32	(AG)_8_YT	21	15	71	0.328
sr33	(AG)_8_T	13	8	62	0.283
sr34	(TC)_8_CC	11	8	73	0.334
sr35	(TC)_8_CG	30	29	97	0.403
sr36	(AC)_8_CG	9	8	89	0.408
sr37	(AC)_8_C	24	20	83	0.343
sr39	(GA)_8_GG	18	14	78	0.357
sr40	(AC)_8_T	24	19	79	0.336
sr41	(AG)_10_C	29	25	86	0.366
sr42	(AG)_8_YA	25	25	100	0.430
sr50	(TC)_9_C	14	12	86	0.394
sr53	(CT)_8_A	12	9	75	0.344
sr55	(CAC)_6_A	13	10	77	0.353
sr56	(CAC)_6_G	12	6	50	0.230
sr58	(ACC)_6_T	19	10	53	0.242
sr60	(CAC)_7_T	17	12	71	0.324
Mean		19	15	79	0.344

### Molecular data analysis

ISSR results were transformed into binary character matrix in the MS Excel by coding the presence of the clear band as 1 and its absence as 0. Marker informativeness was calculated for each primer by counting TNF—total number of fragments, NPF—number of polymorphic fragments and PPF—percentage of polymorphic fragments. PIC value (Polymorphism Information Content) was calculated according to Roldán-Ruiz et al. (2000a, b) as a mean of all PIC values for all amplified fragments using the formula:

$${\text{PIC}} = 2*f_{i} *\left( {1 - f_{i} } \right)$$

- f_i_ frequency of the amplified allele (band present)
- (1 − f_i_) frequency of the null allele (band absent)

The genetic distance between studied genotypes was calculated based on Nei’s formula (Nei and Li 1979). The geographic distance was calculated based on geographic coordinates of collection sites. The Mantel test (Smouse et al. 1986) with 999 permutations was conducted to seek the relationship between geographic and genetic distance. Unbiased expected heterozygosity (He), percentage of polymorphic products as well as private bands were estimated by GenAlex 6.502 (Peakall and Smouse 2012). Analysis of Molecular Variance within and between groups of individuals divided based on the place of gathering, was determined by 999 permutations.

Relationships among all examined genotypes was estimated using the Dice algorithm (1945) and cluster analysis were performed using UPGMA (Unweighted Pair Group Method with Arithmetic Mean) with 1000 bootstraps, as well as PCoA (Principal Coordinate Analysis) in PAST 3.25 (Hammer et al. 2001).

The population structure was investigated using a Bayesian approach in the program STRUCTURE 2.3.4 (Porras-Hurtado et al. 2013). The admixture model (assuming that individuals may have part of the genome from each of the K populations) and correlated allele frequencies between populations were chosen. Run length was given 10,000 burn-ins followed by 100 000 Markov Chain Monte Carlo replications. The number of clusters (K) were set from 2 to 5, each K value was run ten times. To select and visualize the optimal number of clusters, web-based software, StructureSelector (Li and Liu 2018) integrating Clumpak program (Kopelman et al. 2015) was used.

## Results

20 ISSR primers amplified 374 DNA fragments, of which 297 (79%) were polymorphic. In average, a single primer initialized synthesis of 19 fragments (range 9–30). ISSR primer sr35 triggered amplification of the highest number of polymorphic bands (30), whereas sr36—the lowest (9). The rates of informative polymorphic bands varied from 50% (sr56) to 100% (sr42). PIC score ranged from 0.230 (sr56) to 0.430 (sr42) with an average of 0.344 (Table 2).

An average genetic similarity calculated based on Dice algorithm between all analysed samples was 0.635 (0.28–1.00) (data not shown). Genetic similarity indices estimated within Volhynian Polesie (VP) and West Volhynian Upland samples were very high and reached 1.000 and 0.904, respectively (Table 3). The similarity between specimens collected at VP and VU was 0.418 and was nearly the same as genetic similarity of VU samples to *A. arvensis* (0.435). Simultaneously, Dice index value between *A. foemina* from Volhynian Polesie and *A. arvensis* reached 0.283. Pairwise Mantel test showed statistically significant correlation between geographic and genetic distance of all analysed genotypes (0.955, *P* = 0.01).

**Table 3:** Dice genetic similarity of *Anagallis* L. genotypes collected in Volhynian Polesie (VP) and West Volhynian Upland (VU), based on ISSR data

Group	West Volhynian Upland (group 1)	Volhynian Polesie (group 2)
West Volhynian Upland (group 1)	0.904	–
Volhynian Polesie (group 2)	0.418	1
*Anagallis arvensis* L.	0.435	0.283

The AMOVA study found a significant difference (Φ_pt_ = 0.88, *P* = 0.001) between *Anagallis* L. genotypes gathered in Volhynian Polesie (VP) and West Volhynian Upland (VU), Poland. Analysis indicated, that 89% of the variation existed among groups and 11% within groups.

Percentage of polymorphic loci of about 23.8% was observed for *Anagallis* from West Volhynian Upland (VU). Number of private bands for this group was 163. Much lower value (0.08%) was observed for *Anagallis* from Volhynian Polesie. There were 105 bands unique to this group of genotypes (Table 4). Average unbiased expected heterozygosity (uHe) of genotypes groups was 0.091.

**Table 4:** The within-group genetic diversity of *Anagallis foemina* L. genotypes collected in Volhynian Polesie (VP) and West Volhynian Upland (VU), based on ISSR data

Group	Origin	No. of bands	Percentage of polymorphic loci (%)	No. of private bands	Unbiased expected heterozygosity (uHe)
1	West Volhynian Upland	269	23.8	163	0.089
2	Volhynian Polesie	211	0.8	105	0.002
Mean		240	12.3	134	0.091

No. Bands = No. of different bands

No. Private Bands = No. of bands unique to a single population

No. Bands Freq. ≥ 5% = No. of Different Bands with a Frequency ≥ 5%

No. LComm Bands (≤ 25%) = No. of Locally Common Bands (Freq. ≥ 5%) Found in 25% or Fewer Group

No. LComm Bands (≤ 50%) = No. of Locally Common Bands (Freq. ≥ 5%) Found in 50% or Fewer Group

uHe = Unbiased Expected Heterozygosity = (2 N/(2 N − 1)) * He

**Fig. 2:**
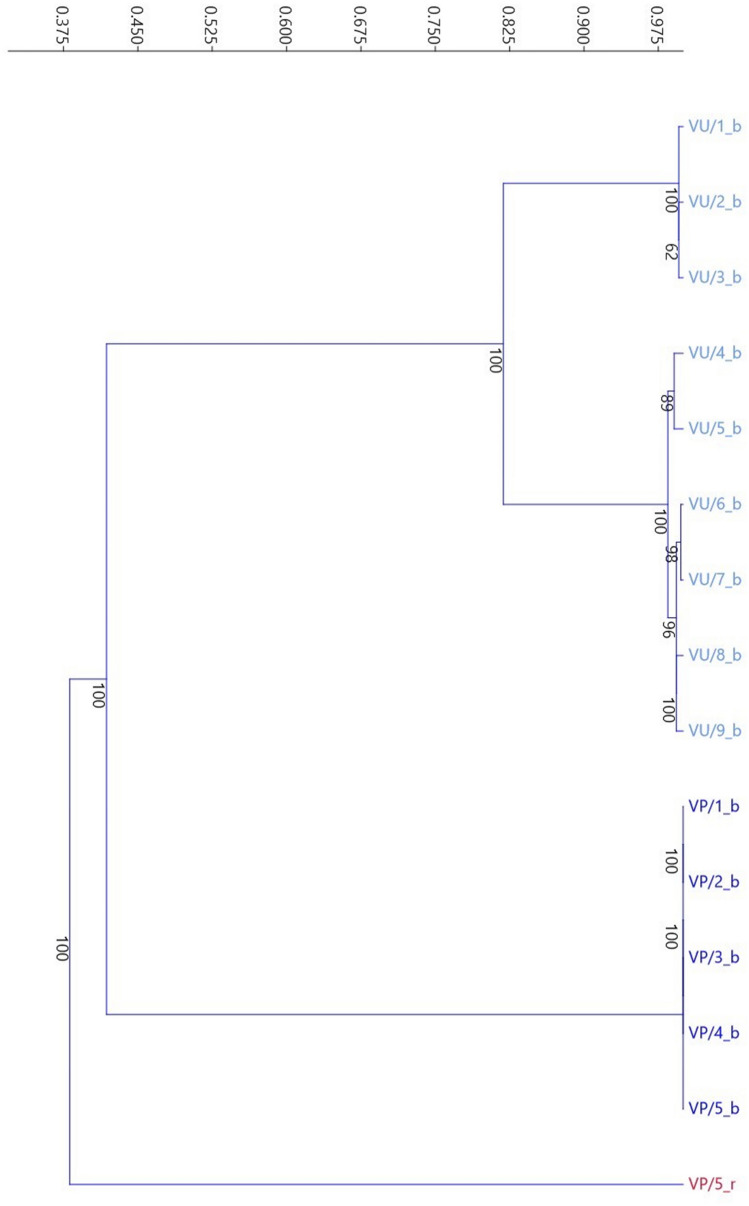
UPGMA dendrogram of *Anagallis* L. genotypes collected in Volhynian Polesie (VP) and West Volhynian Upland (VU), based on Dice similarity index. Bootstrap value indicated on nodes. (_b – *A. foemina*, _r – *A. arvensis*)

**Fig. 3:**
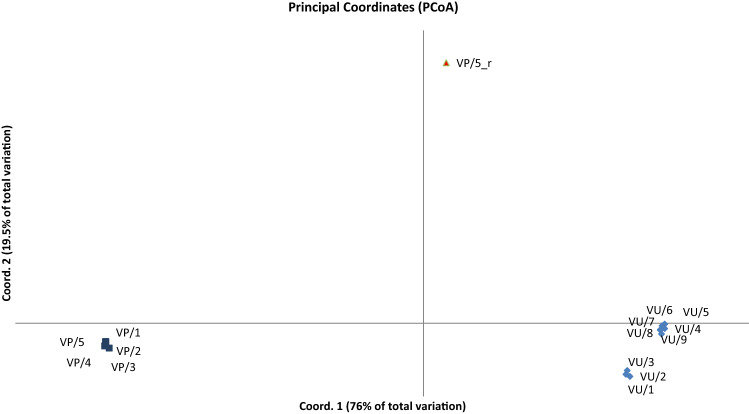
Principal coordinates 1 versus 2 plotted for *Anagallis* L. genotypes collected in Volhynian Polesie (VP) and West Volhynian Upland (VU), Poland, based on Dice similarity index

A single *A. arvensis* genotype was attached to the analysis to verify species affiliation of remaining study objects. UPGMA analyses based on all marker types, grouped *A. foemina* samples into 2 clearly separated clusters (Fig. 2). The first is composed of all *A. foemina* genotypes collected in Volhynian Polesie (VP). Second cluster encompasses *A. foemina* from West Volhynian Upland (VU). Dendrogram shows that *A. arvensis* (VP_r) is distinct from both of these groups and is located separately. Principal coordinate analysis (Fig. 3) gives congruent results, confirming the cluster analyses. The two coordinates explain 95% of total variance.

**Fig. 4:**
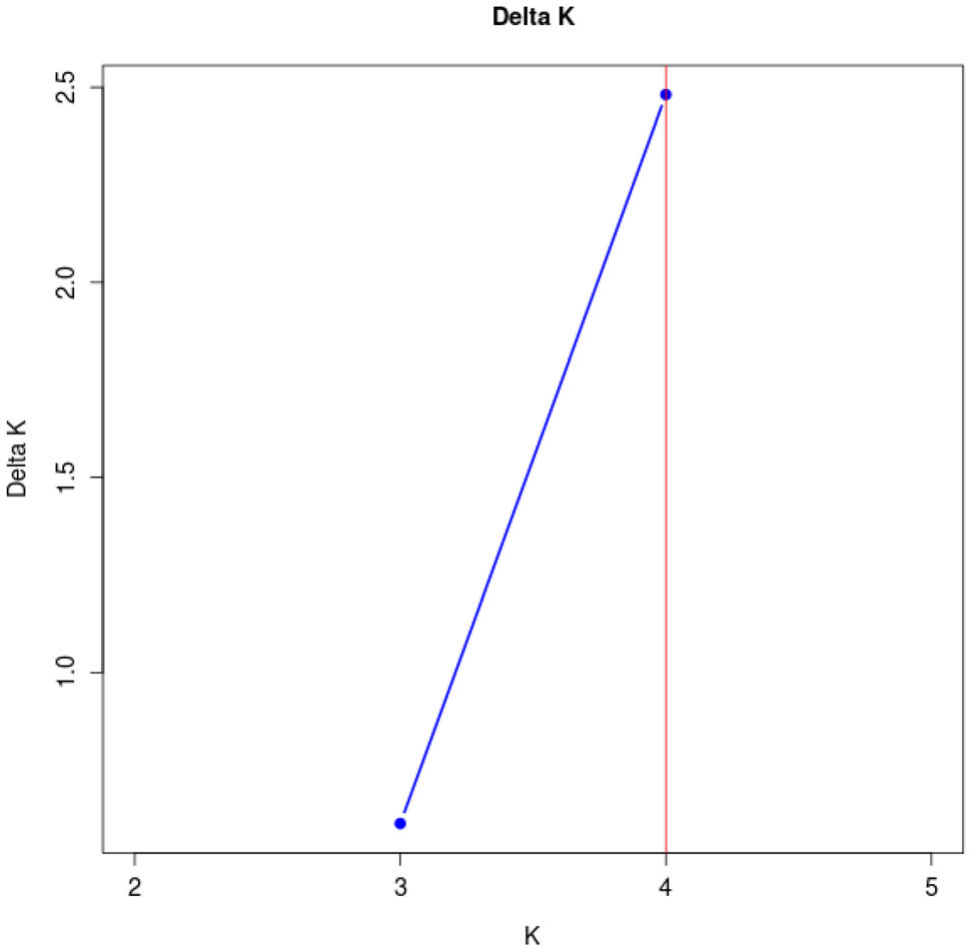
Estimated number of clusters of *Anagallis* L. genotypes obtained for K = 2–5 based on ΔK

**Fig. 5:**
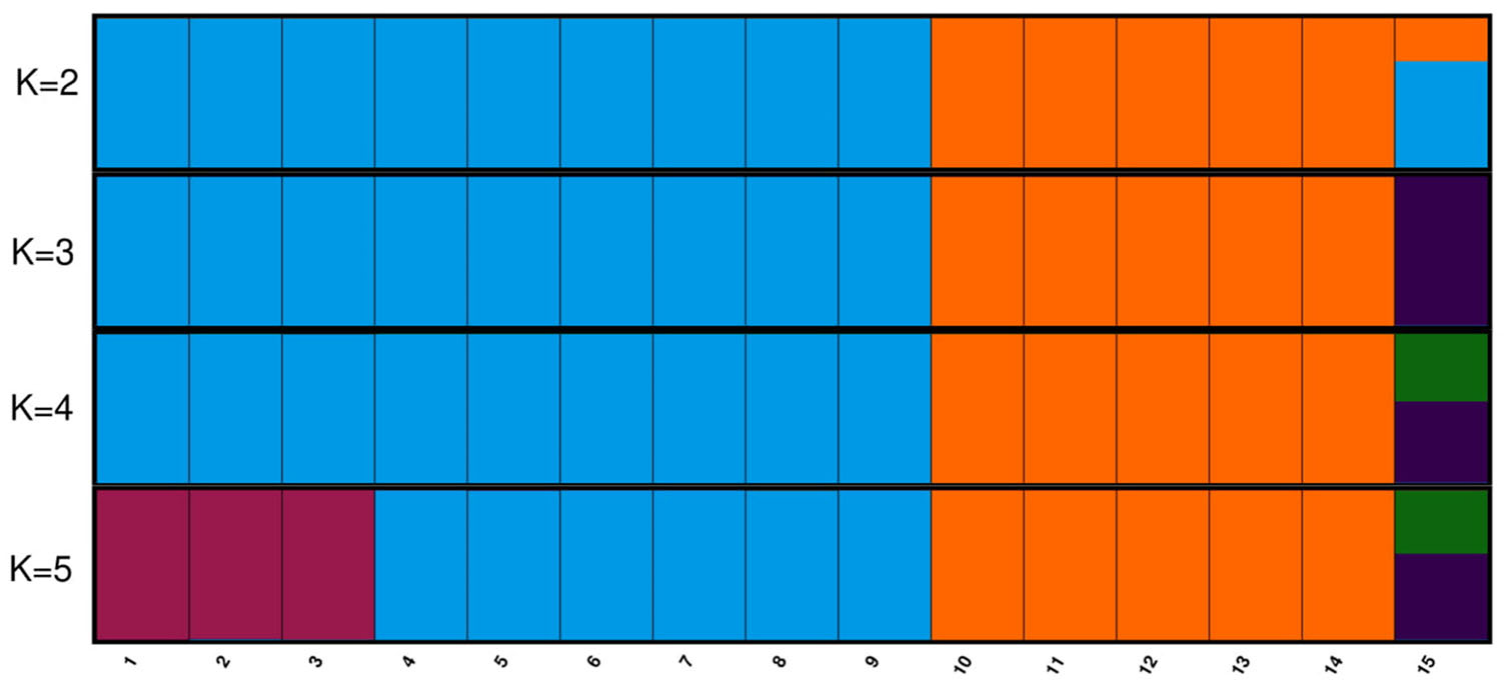
The graphic representation of *Anagallis* L. genotypes structure for K = 2–5. Different colours represent diverse genetic backgrounds. 1–9 VU1–VU9. 10–14 VP1–VP5. 15 VP/5_r (color figure online)

To estimate genetic structure of *Anagallis* L. genotypes, STRUCTURE software was run for K = 2–5. The maximum ΔK occurred at K = 4 and was 2.48150 (Fig. 4). At K = 3, ΔK was equal 0.61182. Remaining results were significantly lower. *A. foemina* collected in West Volhynian Upland (VU) represents first group, second group is composed of *A. foemina* from West Volhynian Upland (VU). *A. arvensis* is recognized as a distinct gene pool (Fig. 5).

## Discussion

Intensification of farming, changes in cultivation techniques, widespread use of plant protection products, thorough cleaning of seeds for sowing and reduction of ruderal habitats area, caused changes in the structure of agrophytocenoses and have an impact on becoming of some weed species rare in plant cultivation (Baessler and Klotz 2006; Storkey et al. 2012; Meyer et al. 2013; Mayerová et al. 2018). Species associated with specific crops and with a narrow ecological amplitude are particularly sensitive to any fluctuations in habitat conditions (Haliniarz and Kapeluszny 2014; Staniak et al. 2017). Archeophytes, growing on heavy calcareous soils, which are species characteristic of the Lathyro-Melandrietum noctiflori, Caucalido-Scandicetum or Kickxietum spuriae complexes are rapidly decreasing from cultivated fields (Nowak et al. 2008). The annual form of weeds makes some species very sensitive to environmental changes and susceptible to population fragmentation (Brütting et al. 2012a, b). Increasingly smaller and fragmented populations of rare species are more sensitive to the threat of loss of genetic diversity, inbred depression or the accumulation of new harmful mutations (Frankham et al. 2002). It is therefore necessary to examine the genetic variability of the population of endangered species, which can be the basis for the development of programs of their protection (Parusel et al. 2009; Frankham 2010; Janiak et al. 2014.

The level of polymorphism revealed by ISSR approach was very high and reached 79%. About 300 polymorphic loci have been identified within analysed materials. According to Nei and Li (1979), 50 differentiating loci are necessary to evaluate the genetic variability. Pejic et al. (1998) reported, that more than 150 markers were necessary to obtain a good precision in analysing genetic diversity, irrespectively of technique employed. Based on PIC values, it can be concluded that marker system capacity to detect polymorphic loci in a single amplification was very efficient as the average value of this coefficient amounted 0.344. This results proved this technique can be conveniently used for the genetic characterization of *Anagallis* species.

Genetic similarity analysis based on Dice algorithm, as well as AMOVA study of *A. foemina* samples gathered from Volhynian Polesie (VP) and West Volhynian Upland (VU) revealed significant difference (Φ_pt_ = 0.88, *P* = 0.001) between genepools derived from these locations. Simultaneously, within groups variation was low (11%) with genetic similarity from 0.904 to 1.000. It indicates a very low diversity of populations, especially Volhynian Polesie derived, and can be an effect of population fragmentation as well as the genetic isolation. Consistent results were obtained by Brütting et al. (2013) who found that *A. foemina* within population diversity was on a very low level (16%). Greater diversity of molecular variance was observed between the populations studied and this is also in agreement with our results. The strong differentiation of *A. foemina* populations between geographical regions indicates that current gene flow is very low. Daco et al. (2019) analyzing the protected species of *Gladiolus palustris*, found that although most of the population of *G. palustris* in the regions retained significant genetic variation, genetic diversity is likely to decrease in small populations due to genetic drift.

UPGMA analyses grouped *A. foemina* samples into 2 clearly separated clusters based on places of their origin, with *A. arvensis* on the edge of dendrogram. PCoA results confirmed the clustering analyses. However the genetic similarity between *A. foemina* populations from Volhynian Polesie and West Volhynian Upland was even lower than genetic similarity of West Volhynian Upland samples to *A. arvensis*. It can indicate that genetic diversity of endangered *A. foemina* species is high and artificial crossing or introduction plants from isolated geographically regions can strengthen the probability of species survival.

A low level of genetic diversity is usually found in small, isolated populations (Jacquemyn et al. 2010; Crichton et al. 2016; Sullivan et al. 2019). The loss of species biodiversity in a given area is one of the main environmental problems (Godefroid et al. 2011; Brütting 2013). This also applies to species that currently survive in small quantities as a result of intensified agriculture (Honnay and Jacquemyn 2007). Such populations are more susceptible to genetic drift and reduced gene flow between populations (Ellstrand and Elam 1993; Sullivan et al. 2019).

More and more species are threatened, including agricultural weeds. Isolation of a small population leads to a decrease in internal genetic variation, which in turn contributes to a decrease in immunity and adaptability to environmental changes, a decrease in reproduction rate and increased mortality. As a consequence of fragmentation, genetic differentiation among populations and the loss of genetic diversity is expected to increase with time. This becomes the reason for the extinction of the local plants populations (Zawko et al. 2001). Hence, assessing the genetic diversity of plants, especially those rare and endangered is so important. A comprehensive approach, including the analysis of anthropogenic and habitat impacts, allows determining the species susceptibility to population reduction and developing strategies for its protection. According to Daco et al. (2019), in the case of high fragmentation of the population, in order to preserve biodiversity, the conservation of populations from each regional cluster is essential.
